# Gender differences in recovery consequences among heroin dependent patients after compulsory treatment programs

**DOI:** 10.1038/srep17974

**Published:** 2015-12-08

**Authors:** Jiang Haifeng, Liang Di, Du Jiang, Sun Haiming, Chen Zhikang, Fu Liming, Zhao Min

**Affiliations:** 1 Shanghai Mental Health Center, Shanghai Jiao Tong University School of medicine, 600 South Wanping Road, Shanghai 200030, PR China; 2 UCLA Fielding School of Public Health, Department of Health Policy and Management, 650 Charles Young Dr. S., 31-269 CHS Box 951772, Los Angeles, CA, 90095-1772, USA; 3 Council of Shanghai Ziqiang Social Services, 158 Hanzhong Road, Shanghai 200070, PR China

## Abstract

Studies on recovery patterns and how baseline factors influence recovery consequences among heroin dependent patients have shown mixed results. This study is aimed at describing the gender differences in long-term recovery patterns and exploring the predictors of negative recovery consequences by gender among heroin dependent patients in Shanghai, China. At baseline, this study recruited 503 heroin dependent patients discharged from Shanghai compulsory rehabilitation facilities in 2007 and 2008. In this cohort study, the baseline data was then linked with participants’ 5-year follow-up data from official records. Generalized Estimating Equations (GEE) were used to compare males with females in terms of the presence of negative consequences (incarceration, or readmission to compulsory treatment, or both), in the subsequent 5-years after their discharge from compulsory treatment. Ordinary least squares (OLS) regression was used to explore factors associated to the time length of negative consequences in 5 years after the discharge for males and females separately. Our findings indicate that female heroin dependent patients tend to have less negative recovery outcomes than male patients. Male patients with a life-time history of poly drug use and female patients with borderline personality disorder are especially at risk of incarceration and readmission into compulsory treatment programs.

Despite the increased use of a wide range of stimulative drugs, the use of opioids continues to be a major drug problem globally^1^,^2^. Obviously heroin dependence is associated with high rates of mortality, morbidity, and other adverse consequences, such as criminal involvement and incarceration^3^,^4^,^5^,^6^. However, studies on recovery patterns and how baseline factors influence recovery consequences among heroin dependent patients have shown mixed results. Moreover, most previous studies focused on the population in Europe and North America, and the population in Asia was largely understudied.

In this study, we are especially interested in gender differences in recovery. Past researches have shown that men and women differ in treatment processes, retention, completion, outcomes, and determinants of post-treatment outcomes^7^,^8^,^9^. Such variations may be explained by gender differences in social supports and mental health status^6^,^10^,^11^. However, previous studies on the gender effects on long-term recovery consequences have revealed mixed results^12^,^13^. Predictors for recovery consequences by gender were also understudied among population in Asia. Thus, studies on the gender effects, especially in diverse settings and cultural backgrounds, are still in need.

According to the Anti-Drug Law of the People’s Republic of China which was issued in 2007, illegal drug users discharged from compulsory treatment programs should receive community recovery for around two years^14^,^15^. The lack of empirical data on patterns of relapse and criminal activities and predictors to recovery consequence among Chinese patient population has become one major challenge to evaluate and improve the recovery model, particularly when China shift to a new recovery model and system. To date, the recovery pattern and associated factors among Chinese population who evolved in the new model has not been reported before.

In order to fill the knowledge gaps discussed above, this study obtained longitudinal data on a group of heroin dependent patients who were discharged from compulsory treatment facilities in Shanghai. This study examined how males and females differ in recovery patterns and predictors of negative consequences (re-use and criminality) from heroin addiction. We hope that better understanding of how females and males differ in their recovery patterns and the influential factors can contribute to improving gender-appropriate interventions and recovery strategies.

## Results

### Descriptive analysis

On average, heroin dependent patients in our study had a baseline history of 2.6 times of compulsory treatment and 10.7 months of incarceration. The analytic sample included 238 males and 265 females. Of all participants, the mean age was 26.4 years old. Most of them were Han, more than one-half (54.1%) had a less than high school education, about one-fourth of the sample (29.2%) were currently married, and less than one-third (29.8%) were employed before admission. Prior to being admitted to compulsory treatment, the reported average daily heroin dose of the participants was 0.84 gram for males and 1.04 gram for females. In terms of mental health status of participants, 14.3% of participants reported a life-time history of any mental disorder (excluding other illegal drug use), 18.7% reported a life-time history of Borderline Personality Disorder, and 32.8% reported a life-time history of Antisocial Personality Disorder. Table 1 presents the demographic and clinical characteristics of the participants.

### Recovery patterns

**Figure 1:**
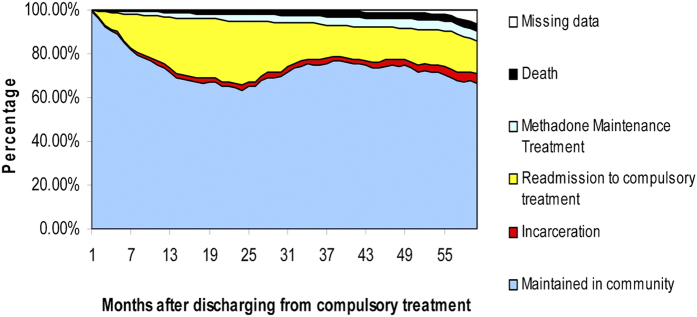
Natural history of heroin dependent patients who discharged from the compulsory treatment program in Shanghai, China.

**Figure 2:**
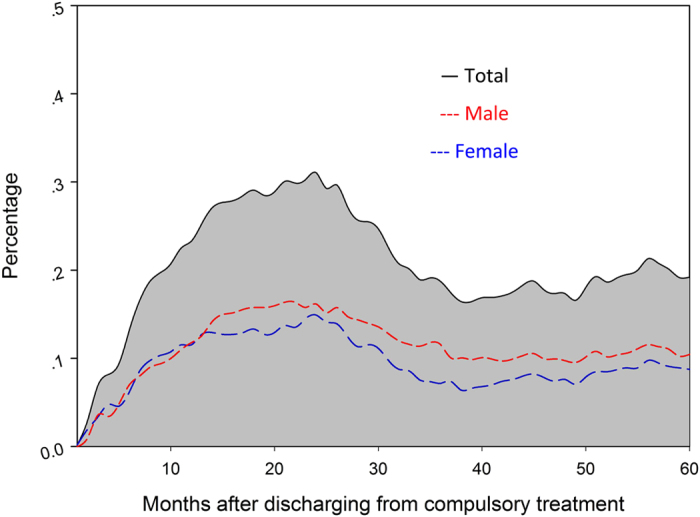
Percentage of male and female participants who having negative consequences in 5 years after the discharge from compulsory treatment.

To examine the cohort’s natural history of heroin use, we have graphically displayed the sample’s entire recovery pattern after discharge from compulsory treatment programs (Fig. 1). Initial GEE analyses based on 5 years (60 months) longitudinal data showed the odds of having negative consequences in 5 years after the discharge from compulsory treatment among females are 0.811 times of the odds of males (Fig. 2 and Table 2).

### Predictors to negative outcomes

OLS analyses were conducted for males and females separately. After excluding death cases and adjusting age, education differences, results showed that having a life-time history of using other illegal drugs was associated with having 6.8 more months of negative outcomes among males (95% CI: 1.1-12.5, p < 0.05); have a life-time history of Borderline Personality Disorder was associated with having 7.0 months of negative outcomes among females (95% CI: 2.0-12.0, p < 0.05) (Table 3).

## Discussion

This study examined gender differences in recovery patterns among a sample of patients discharged from compulsory treatment over a five-year period in Shanghai, China. The present study also suggested that baseline predictors of incarceration and readmission to compulsory treatment at post-treatment follow-up were different for male and female. This is the first study that has used longitudinal data to explore gender difference among a Chinese population. These findings also extended our knowledge of gender difference in longitudinal patterns of recovery and predictors for negative consequence.

Foremost among the findings is the confirmation that the longitudinal patterns of recovery differ by gender. After adjusting life-time history of mental disorders, demographic characteristics, and baseline addiction severity, this study still found a significant gender differences in longitudinal patterns of recovery among Chinese heroin dependent patients. This finding suggests that except for those known factors, there are perhaps some additional factors which may potentially mediate or moderate the effects of gender. However, the underlying mechanisms of gender effects still could not be identified in this study, as in most previous studies. Future studies can focus on those unknown factors which may mediate or moderate gender’s effects on recovery consequences. Future studies that have analysis with those potential factors will help to explore whether the gender per se or other factors correlated with gender are the key drivers of gender difference in recovery.

The significant predictor at baseline for higher risk of negative consequences was using multiple drugs for men. This result is consistent with previous studies^16^. Thus, we suggest healthcare providers to pay special attention to heroin dependent patients who use multiple substances, especially among males. For women, the significant predictor at baseline is having a life-time history of Borderline Personality Disorder (BPD). Previous studies revealed that having a co-occurring mental illness is associated with worse substance abuse treatment outcomes^16^,^17^, and more crime involvements^16^,^18^. Although more males reported to have a life-time history of BPD at baseline than females, this factor was not a significant predictor for male’s outcome. Since co-morbidity of personality disorder and substance use was common among heroin addicts in Chinese compulsory treatment programs^19^, our finding highlight the importance of integrating psychological intervention in compulsory treatment for females to achieve optimal recovery outcomes. Different from findings reported in the literature^16^,^20^,^21^,^22^, age, marriage, education, employment, life-time history of antisocial personality disorder, and life-time history of depression or anxiety were not significant predictors to post-treatment negative consequences.

The study findings should be considered within the context of the limitations. First, due to limitations of the data, we were not able to check the abstinence status of each participant when they were in MMT or in the community. Some patients may relapse but were not found or sent to a compulsory treatment center. Thus, we were not able to estimate patients’ relapse status using our outcome “negative consequences” in the study. Future studies may consider using urine tests to verify heroin dependent patients’ drug use status in the community. Second, all samples were recruited from the compulsory treatment programs in Shanghai. Thus, our results might not be generalized to heroin dependent patients who have never entered a compulsory treatment program or from other areas of China.

In summary, our study explored the gender differences in longitudinal recovery patterns and predictors for negative consequences after being discharged from compulsory treatment programs. This information can contribute to improving gender-appropriate interventions and recovery strategies.

## Methods

### Study design and Data Sources

This study was designed as a cohort study. After the baseline assessment, we passively followed up our participants and ascertained their long-term recovery consequences using participants’ official record archives. The baseline data was collected from the project “Five years follow-up study on heroin dependent patients in Shanghai.” This study was funded by Shanghai Municipal Narcotics Control Committee to assess the longitudinal patterns of heroin use and HIV risk behaviors among heroin users in Shanghai, China. The follow-up data was extracted from an electronic monthly summary record system, initially built by Shanghai Municipal Narcotics Control Committee in March 2007. This electronic database was managed by social workers who are employed by the government to help drug users in the community, and to monitor drug use behaviors. This official electronic database now contains the following information of over 40,000 registered drug users: ID number, basic demographic information, legal status, and drug abuse treatment history. The unique ID number was used to link our baseline data to the official record database.

Each participant’s 5-year follow-up data was extracted from the official record database from 2008 to 2013 since their discharge from compulsory treatment centers. In the official record database, the following categories of drug-related information were recorded each month in a chronological order: incarceration, readmission to compulsory treatment, methadone maintenance treatment participation. These events were recorded as binomial variables (happened or not).

This study was approved by the institutional Review Boards in Shanghai Mental Health Center. Informed consent was obtained from all participants. All procedures were in accordance with the approved guidelines.

### Setting and Participants

The baseline study used convenience sampling to recruit any patients who met study inclusion criteria and were mandated to undergo drug rehabilitation programs. After informed consent, participants would be followed for 5 years. The baseline study recruited 503 heroin dependent patients from three compulsory rehabilitation centers in Shanghai during April of 2007 to May of 2008 according to the following criteria: (a) meeting the fourth edition of the Diagnostic and Statistical Manual of Mental Disorders (DSM-IV) diagnostic criteria for heroin dependence; (b) being at least 18 years of age; (c) having heroin use during the 30 days prior to admission to a rehabilitation program; and (d) having the cognitive capacity to give consent. The present study included all 503 participants from the baseline project.

### Variables and Measurement

At the baseline, a Chinese version of the Addiction Severity Index (ASI) was used to assess the social demographic characteristics and history of drug abuse. The reliability and validity of the Chinese version of the instrument has also been assessed^23^,^24^: the internal consistency of the seven dimensions is acceptable (alpha = 0.44 ~ 0.79), the test-retest reliability is good (ICC = 0.68 ~ 0.84), and the inter-rater reliability is good (ICC = 0.87 ~ 0.98). Mental health and personality disorder were diagnosed with the Structured Clinical Interview for DSM-IV (SCID Axis I and II Disorders) by trained psychiatrists.

For the present study, the recovery statue of each month was classified into 5 outcomes: incarceration, readmission to compulsory treatment, methadone maintenance treatment participation, death, and maintained in community (Not encoded as anyone of other cases). Our outcome variable of interest, ‘negative consequence’, is defined as having incarceration or readmission to compulsory treatment or both. According to laws in China, an illegal drug use case will be readmitted to compulsory treatment but not be defined as incarceration. In compulsory treatment centers, drug users will receive detoxification, education, labor work, etc. However, we consider readmission to compulsory treatment as negative outcome during community recovery, because readmission was only triggered by seriously violating the community recovery agreement or re-using drugs after compulsory treatment, which means that those readmission cases relapsed or had other serious consequences.

### Statistical methods

We used Stata 12^25^ for our data analysis. Gender differences in baseline characteristics were assessed using chi-square statistics for categorical variables and t test for continuous variables. Generalized Estimating Equations (GEE) was used to compare males with females in terms of the presence of negative outcomes (incarceration and readmission to compulsory treatment) in the subsequent 5 years after the discharge from compulsory treatment. In the GEE model, as our outcome variable is binary, we specified the model using the binomial distribution function and a logit link function with identity covariance matrix. The following covariates were included in the model: life-time history of mental disorders (anxiety disorder, mood disorders, and psychotic disorders), the life-time history of using illicit drugs other than heroin, the life-time history of borderline personality disorder and antisocial personality disorder, education, baseline employment status, baseline marriage status, age of onset of using heroin, baseline age, baseline dose of heroin use, baseline frequency of heroin use, and incarceration and compulsory treatment experience before baseline. The interaction between gender and time effect was also examined in the model. In addition, we also used ordinary least squares (OLS) to explore factors associated to the time length of negative outcomes in 5 years after the discharge for both gender. The significance level for all statistical tests was set at *p* < 0.05 (two-tailed).

## Additional Information

**How to cite this article**: Haifeng, J. *et al.* Gender differences in recovery consequences among heroin dependent patients after compulsory treatment programs. *Sci. Rep.* **5**, 17974; doi: 10.1038/srep17974 (2015).

## Figures and Tables

**Table 1:** The demographic and clinical characteristics of the participants.

	Total (N = 503)	Male (n = 238)	Female (n = 265)
**Age**, mean ± s.d., years	26.4 ± 7.9	27.8 ± 8.5	25.2 ± 7.1
**Ethnicity**, n (%)			
Han	497(98.8%)	235(98.7%)	262 (98.9%)
**Education**, n (%)			
Less than high school	272 (54.1%)	143 (60.1%)	129 (48.7%)
High school (9–12 grade)	208 (41.3%)	85 (35.7%)	123 (46.4%)
More than high school	23 (4.6%)	10 (4.2%)	13 (4.9%)
**Marital status**, n (%)			
Currently married	147 (29.2%)	57 (23.9%)	90 (34.0%)
**Employment status in the 3 years prior to the baseline interview**, n (%)			
Employed (fulltime & part time & housewife)	150 (29.8%)	72 (30.3%)	78 (29.4%)
unemployed	322 (64.0%)	139 (58.4%)	183 (69.1%)
incarcerated	31 (6.2%)	27 (11.3%)	4 (1.5%)
**Onset age,** mean ± s.d., years	23.6 ± 7.9	24.5 ± 7.1	22.7 ± 7.5
**Main way of drug use**, n (%)			
Injection	29 (5.8%)	8 (3.4%)	21 (7.9%)
Sniffing	449 (89.3%)	219 (92.0%)	230 (86.8%)
unknown	25 (5.0%)	11 (4.6%)	14 (5.3%)
**Daily dose of heroin before the baseline interview (n = 493**), mean ± s.d., gram	0.95 ± 0.55	0.84 ± 0.56	1.04 ± 0.52
**Mean No. of compulsory rehabilitation before the baseline interview (n = 499)**, mean ± s.d.	2.6 ± 1.7	2.7 ± 1.7	2.4 ± 1.6
**Incarceration time before the baseline interview in month (n = 498)**, mean ± s.d.	10.7 ± 25.5	20.3 ± 33.5	2.0 ± 8.6
**Life-time history of any mental disorder and other illegal drug use**, n (%)	170 (33.8%)	61 (25.6%)	109 (41.1%)
**Life-time history of any mental disorder (excluding other illegal drug use)**, n (%)	72 (14.31%)	30 (12.6%)	42 (15.9%)
**Life-time history of Psychosis**, n (%)	27 (5.4%)	10 (4.2%)	17 (6.4%)
**Life-time history of Anxiety**, n (%)	18 (3.6%)	9 (3.8%)	9 (3.4%)
**Life-time history of Mood disorder**, n (%)	37 (7.4%)	16 (6.7%)	21 (7.9%)
**Life-time history of Borderline Personality Disorder**, n (%)	94 (18.7%)	55 (23.1%)	39 (14.7%)
**Life-time history of Antisocial Personality Disorder**, n (%)	165 (32.8%)	127 (53.4%)	38 (14.3%)

**Table 2:** Comparison of recovery patterns after discharge from compulsory treatment by gender using GEE (n = 466^+^).

	Main effects of being female	Main effects of Time	Interaction effects of being female × Time
Negative consequences* after adjustment	OR = 0.811 CI:[0.715, 0.920] (*p* = 0.001)	OR = 1.004 CI:[1.002, 1.006] (*p* = 0.001)	OR = 0.994 CI:[0.991, 1.006] (*p* = 0.001)
Negative consequences* without adjustment^	OR = 0.807 CI:[0.719, 0.905] (*p* < 0.001)	OR = 1.004 CI:[1.002, 1.006] (*p* = 0.001)	OR = 0.994 CI:[0.991, 0.997] (*p* = 0.001)

*Negative consequences: defined as having incarceration or readmission to compulsory treatment or both. ^Adjusted covariates: life-time history of mental disorders (anxiety disorder, mood disorders, and psychotic disorders), the life-time history of using illicit drugs other than heroin, the life-time history of borderline personality disorder and antisocial personality disorder, education, baseline employment status, baseline marriage status, age of onset of using heroin, baseline age, baseline dose of heroin use, baseline frequency of heroin use, and incarceration and compulsory treatment experience before baseline. ^+^16 Death cases of total participants were excluded in the analysis. 21 cases were excluded automatically by Stata 12 due to missing data in covariates.

**Table 3:** Risk factors associated with the number of months in incarceration and compulsory treatment during 5 years after discharge from compulsory treatment for both gender estimated by OLS model.

	Male (n = 214), Mean Difference (95% CI)	Female (n = 252), Mean Difference (95% CI)
Marriage (binary)	0.60 (−5.58, 4.38)	−1.12 (−4.84, 2.60)
Baseline age	−0.12 (−0.68, 0.44)	0.05 (−0.26, 0.36)
Education		
Less than high school	0.56 (−9.59, 10.71)	4.11 (−4.24, 12.45)
high school	−3.63 (−13.87, 6.61)	3.08 (−5.15, 11.31)
more than high school	Reference	Reference
Employment (binary)	0.06 (−4.87, 4.99)	−2.62 (−6.39, 1.14)
Drug use history		
Onset age	0.33 (−0.27, 0.94)	−0.16 (−0.38, 0.05)
Current daily frequency of drug use each day	−0.06 (−1.51, 1.38)	0.23 (−0.97, 1.44)
Current daily dose of drug use each day	−2.05 (−5.97, 1.87)	0.07 (−3.54, 3.67)
No. of compulsory rehabilitation before the baseline interview	0.11 (−1.47, 1.24)	−0.73 (−1.88, 0.43)
No. of month for incarceration before the baseline interview	0.02 (−0.05, 0.08)	−0.13 (−0.33, 0.06)
Life-time history of using other illegal drugs (binary)	6.79 (1.06, 12.51)*	1.06 (−2.65, 4.76)
Mental health history		
Life-time history of having psychosis (binary)	−1.83 (−12.75, 9.09)	6.45 (−0.63, 13.53)
Life-time history of having anxiety disorder (binary)	−6.09 (−17.17, 4.99)	−6.16 (−16.16, 3.84)
Life-time history of having mood disorders (binary)	−2.19 (−11.33, 6.95)	0.59 (−5.49, 6.67)
Life-time history of having borderline personality disorder (binary)	−2.42 (−7.79, 2.95)	6.98 (2.02, 11.95)**
Life-time history of having antisocial personality disorder (binary)	−0.47 (−4.94, 3.99)	−1.74 (−6.99, 3.50)

*p = 0.020; **p = 0.006
